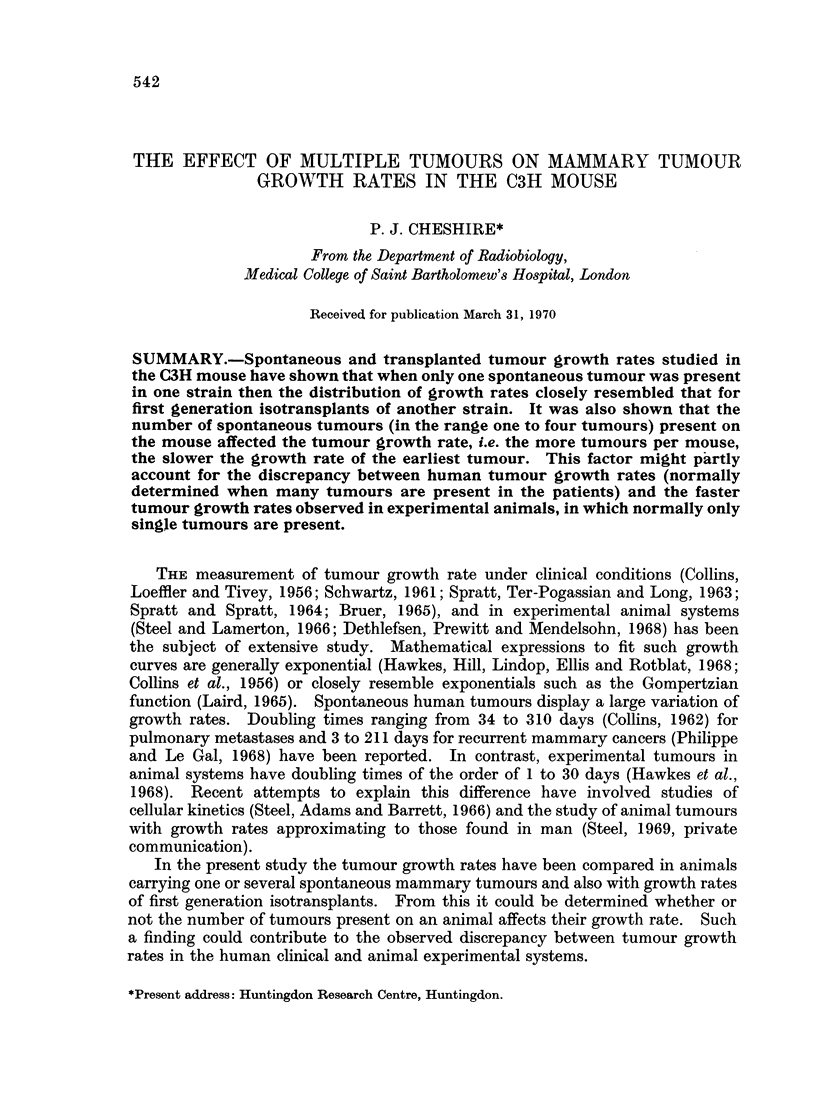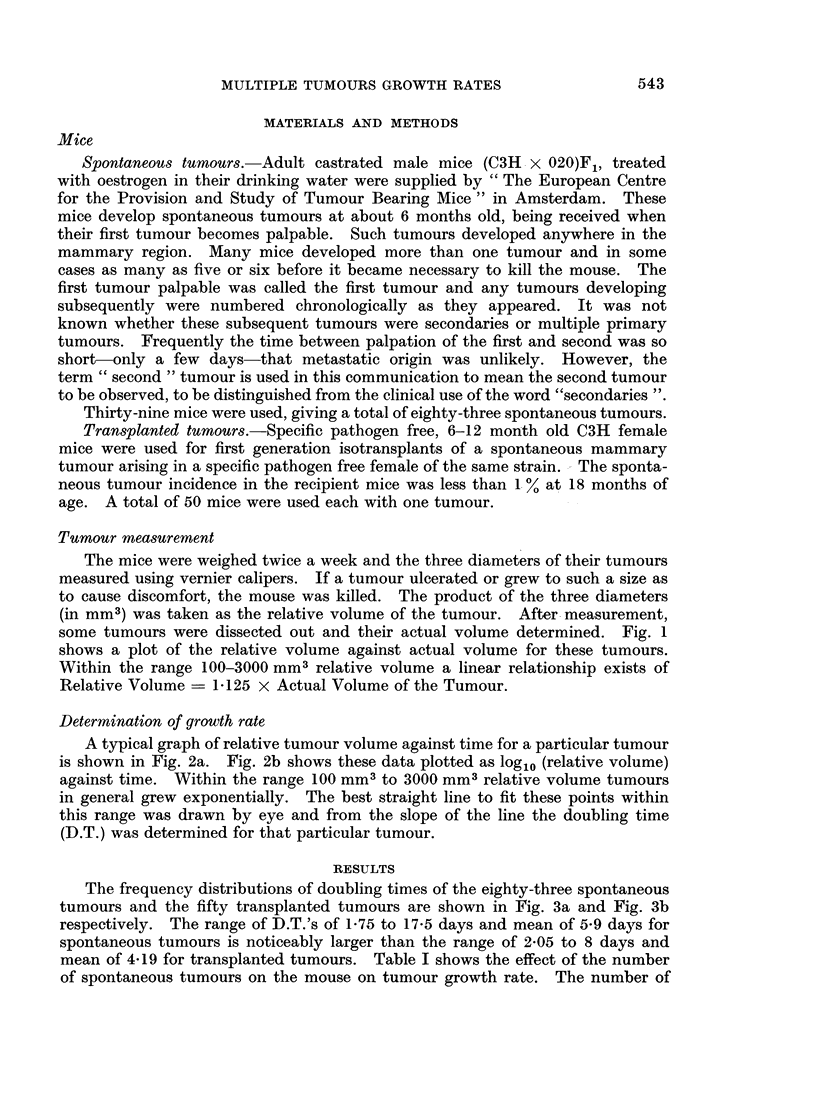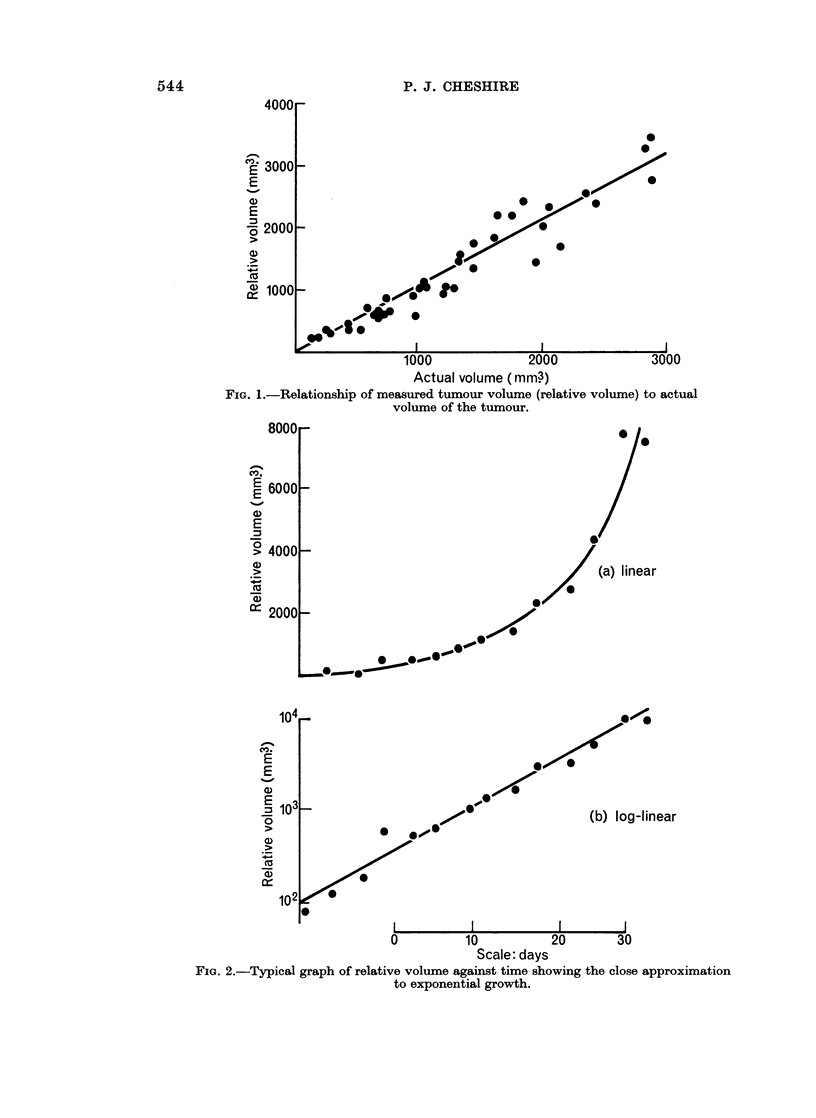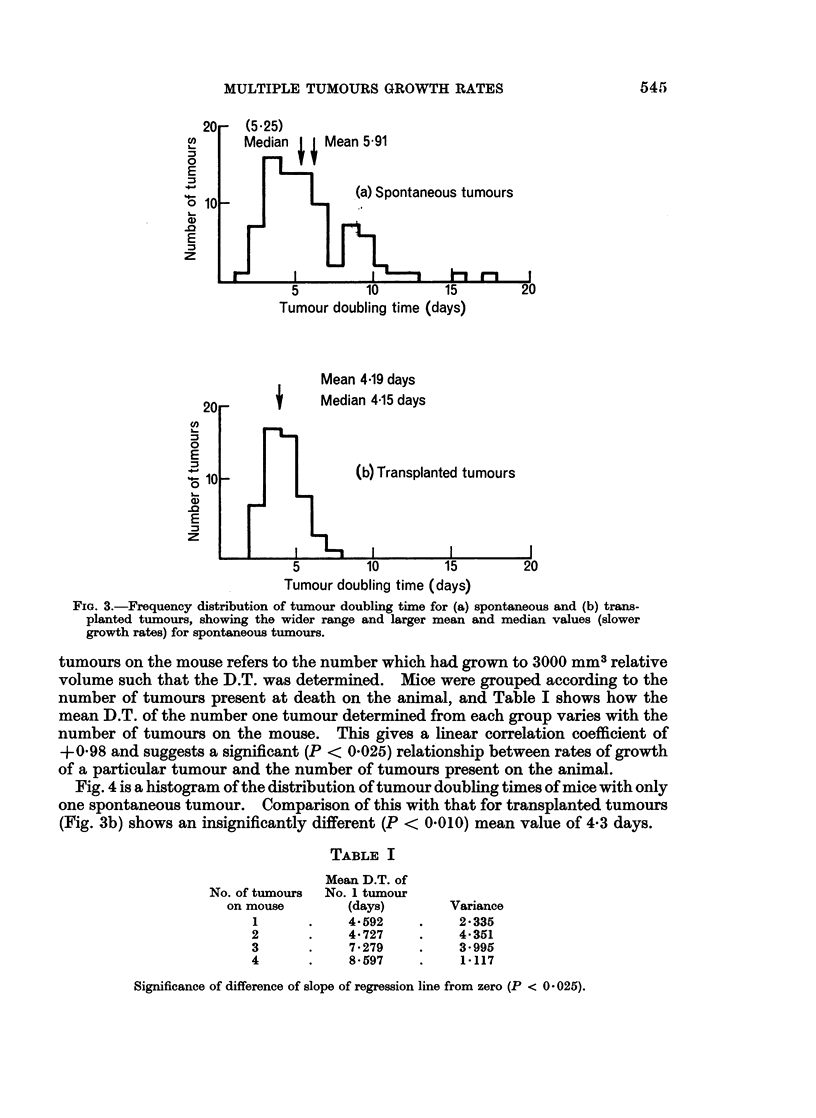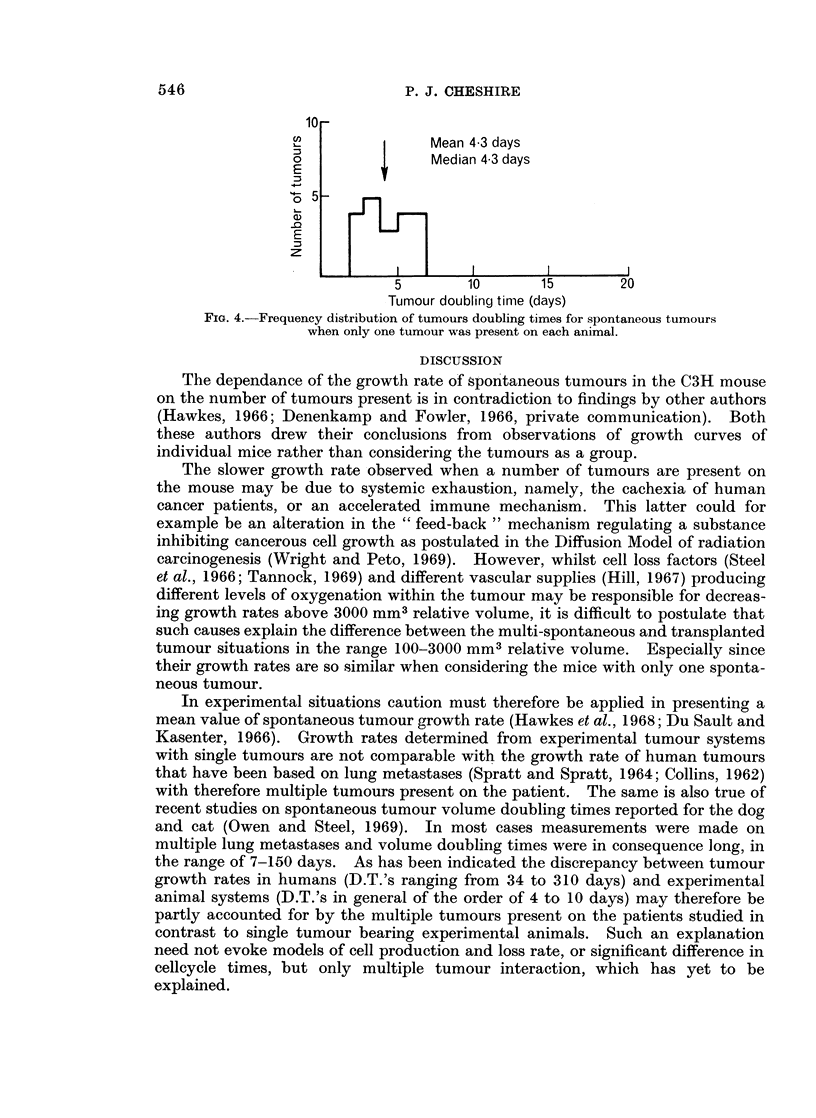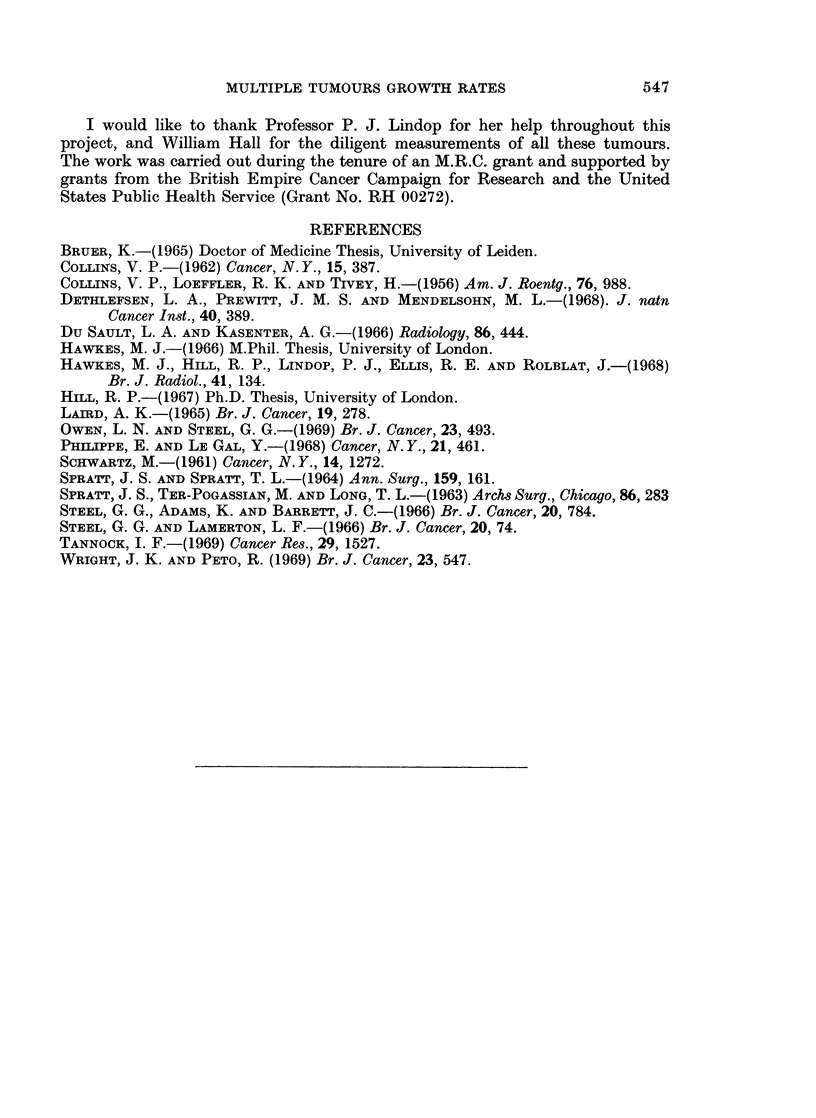# The Effect of Multiple Tumours on Mammary Tumour Growth Rates in the C3H Mouse

**DOI:** 10.1038/bjc.1970.65

**Published:** 1970-09

**Authors:** P. J. Cheshire

## Abstract

Spontaneous and transplanted tumour growth rates studied in the C3H mouse have shown that when only one spontaneous tumour was present in one strain then the distribution of growth rates closely resembled that for first generation isotransplants of another strain. It was also shown that the number of spontaneous tumours (in the range one to four tumours) present on the mouse affected the tumour growth rate, *i.e.* the more tumours per mouse, the slower the growth rate of the earliest tumour. This factor might partly account for the discrepancy between human tumour growth rates (normally determined when many tumours are present in the patients) and the faster tumour growth rates observed in experimental animals, in which normally only single tumours are present.


### THE EFFECT OF MULTIPLE TUMOURS ON MAMMARY TUMOUR GROWTH RATES IN THE C3H MOUSE

P. J. CHESHIRE*

*From the Department of Radiobiology,*
*Medical College of Saint Bartholomew's Hospital, London*

Received for publication March 31, 1970

**SUMMARY.—Spontaneous and transplanted tumour growth rates studied in the C3H mouse have shown that when only one spontaneous tumour was present in one strain then the distribution of growth rates closely resembled that for first generation isotransplants of another strain. It was also shown that the number of spontaneous tumours (in the range one to four tumours) present on the mouse affected the tumour growth rate, *i.e.* the more tumours per mouse, the slower the growth rate of the earliest tumour. This factor might partly account for the discrepancy between human tumour growth rates (normally determined when many tumours are present in the patients) and the faster tumour growth rates observed in experimental animals, in which normally only single tumours are present.**

THE measurement of tumour growth rate under clinical conditions (Collins, Loeffler and Tivey, 1956; Schwartz, 1961; Spratt, Ter-Pogassian and Long, 1963; Spratt and Spratt, 1964; Bruer, 1965), and in experimental animal systems (Steel and Lamerton, 1966; Dethlefsen, Prewitt and Mendelsohn, 1968) has been the subject of extensive study. Mathematical expressions to fit such growth curves are generally exponential (Hawkes, Hill, Lindop, Ellis and Rotblat, 1968; Collins *et al.*, 1956) or closely resemble exponentials such as the Gompertzian function (Laird, 1965). Spontaneous human tumours display a large variation of growth rates. Doubling times ranging from 34 to 310 days (Collins, 1962) for pulmonary metastases and 3 to 211 days for recurrent mammary cancers (Philippe and Le Gal, 1968) have been reported. In contrast, experimental tumours in animal systems have doubling times of the order of 1 to 30 days (Hawkes *et al.*, 1968). Recent attempts to explain this difference have involved studies of cellular kinetics (Steel, Adams and Barrett, 1966) and the study of animal tumours with growth rates approximating to those found in man (Steel, 1969, private communication).

In the present study the tumour growth rates have been compared in animals carrying one or several spontaneous mammary tumours and also with growth rates of first generation isotransplants. From this it could be determined whether or not the number of tumours present on an animal affects their growth rate. Such a finding could contribute to the observed discrepancy between tumour growth rates in the human clinical and animal experimental systems.

*Present address: Huntingdon Research Centre, Huntingdon.

### MATERIALS AND METHODS

### *Mice*

*Spontaneous tumours.*—Adult castrated male mice (C3H × 020)$F_1$, treated with oestrogen in their drinking water were supplied by " The European Centre for the Provision and Study of Tumour Bearing Mice " in Amsterdam. These mice develop spontaneous tumours at about 6 months old, being received when their first tumour becomes palpable. Such tumours developed anywhere in the mammary region. Many mice developed more than one tumour and in some cases as many as five or six before it became necessary to kill the mouse. The first tumour palpable was called the first tumour and any tumours developing subsequently were numbered chronologically as they appeared. It was not known whether these subsequent tumours were secondaries or multiple primary tumours. Frequently the time between palpation of the first and second was so short—only a few days—that metastatic origin was unlikely. However, the term " second " tumour is used in this communication to mean the second tumour to be observed, to be distinguished from the clinical use of the word "secondaries ".

Thirty-nine mice were used, giving a total of eighty-three spontaneous tumours.

*Transplanted tumours.*—Specific pathogen free, 6–12 month old C3H female mice were used for first generation isotransplants of a spontaneous mammary tumour arising in a specific pathogen free female of the same strain. The spontaneous tumour incidence in the recipient mice was less than 1 % at 18 months of age. A total of 50 mice were used each with one tumour.

### *Tumour measurement*

The mice were weighed twice a week and the three diameters of their tumours measured using vernier calipers. If a tumour ulcerated or grew to such a size as to cause discomfort, the mouse was killed. The product of the three diameters (in $mm^3$) was taken as the relative volume of the tumour. After measurement, some tumours were dissected out and their actual volume determined. Fig. 1 shows a plot of the relative volume against actual volume for these tumours. Within the range 100–3000 $mm^3$ relative volume a linear relationship exists of Relative Volume = 1·125 × Actual Volume of the Tumour.

### *Determination of growth rate*

A typical graph of relative tumour volume against time for a particular tumour is shown in Fig. 2a. Fig. 2b shows these data plotted as $\log_{10}$ (relative volume) against time. Within the range 100 $mm^3$ to 3000 $mm^3$ relative volume tumours in general grew exponentially. The best straight line to fit these points within this range was drawn by eye and from the slope of the line the doubling time (D.T.) was determined for that particular tumour.

### RESULTS

The frequency distributions of doubling times of the eighty-three spontaneous tumours and the fifty transplanted tumours are shown in Fig. 3a and Fig. 3b respectively. The range of D.T.'s of 1·75 to 17·5 days and mean of 5·9 days for spontaneous tumours is noticeably larger than the range of 2·05 to 8 days and mean of 4·19 for transplanted tumours. Table I shows the effect of the number of spontaneous tumours on the mouse on tumour growth rate. The number of

FIG. 1.—Relationship of measured tumour volume (relative volume) to actual volume of the tumour.

FIG. 2.—Typical graph of relative volume against time showing the close approximation to exponential growth.

FIG. 3.—Frequency distribution of tumour doubling time for (a) spontaneous and (b) transplanted tumours, showing the wider range and larger mean and median values (slower growth rates) for spontaneous tumours.

tumours on the mouse refers to the number which had grown to 3000 mm$^3$ relative volume such that the D.T. was determined. Mice were grouped according to the number of tumours present at death on the animal, and Table I shows how the mean D.T. of the number one tumour determined from each group varies with the number of tumours on the mouse. This gives a linear correlation coefficient of $+0{\cdot}98$ and suggests a significant ($P < 0{\cdot}025$) relationship between rates of growth of a particular tumour and the number of tumours present on the animal.

Fig. 4 is a histogram of the distribution of tumour doubling times of mice with only one spontaneous tumour. Comparison of this with that for transplanted tumours (Fig. 3b) shows an insignificantly different ($P < 0{\cdot}010$) mean value of 4·3 days.

TABLE I

| No. of tumours on mouse | Mean D.T. of No. 1 tumour (days) | Variance |
|---|---|---|
| 1 | 4·592 | 2·335 |
| 2 | 4·727 | 4·351 |
| 3 | 7·279 | 3·995 |
| 4 | 8·597 | 1·117 |

Significance of difference of slope of regression line from zero ($P < 0{\cdot}025$).

FIG. 4.—Frequency distribution of tumours doubling times for spontaneous tumours when only one tumour was present on each animal.

### DISCUSSION

The dependance of the growth rate of spontaneous tumours in the C3H mouse on the number of tumours present is in contradiction to findings by other authors (Hawkes, 1966; Denenkamp and Fowler, 1966, private communication). Both these authors drew their conclusions from observations of growth curves of individual mice rather than considering the tumours as a group.

The slower growth rate observed when a number of tumours are present on the mouse may be due to systemic exhaustion, namely, the cachexia of human cancer patients, or an accelerated immune mechanism. This latter could for example be an alteration in the " feed-back " mechanism regulating a substance inhibiting cancerous cell growth as postulated in the Diffusion Model of radiation carcinogenesis (Wright and Peto, 1969). However, whilst cell loss factors (Steel *et al.*, 1966; Tannock, 1969) and different vascular supplies (Hill, 1967) producing different levels of oxygenation within the tumour may be responsible for decreasing growth rates above 3000 $mm^3$ relative volume, it is difficult to postulate that such causes explain the difference between the multi-spontaneous and transplanted tumour situations in the range 100–3000 $mm^3$ relative volume. Especially since their growth rates are so similar when considering the mice with only one spontaneous tumour.

In experimental situations caution must therefore be applied in presenting a mean value of spontaneous tumour growth rate (Hawkes *et al.*, 1968; Du Sault and Kasenter, 1966). Growth rates determined from experimental tumour systems with single tumours are not comparable with the growth rate of human tumours that have been based on lung metastases (Spratt and Spratt, 1964; Collins, 1962) with therefore multiple tumours present on the patient. The same is also true of recent studies on spontaneous tumour volume doubling times reported for the dog and cat (Owen and Steel, 1969). In most cases measurements were made on multiple lung metastases and volume doubling times were in consequence long, in the range of 7–150 days. As has been indicated the discrepancy between tumour growth rates in humans (D.T.'s ranging from 34 to 310 days) and experimental animal systems (D.T.'s in general of the order of 4 to 10 days) may therefore be partly accounted for by the multiple tumours present on the patients studied in contrast to single tumour bearing experimental animals. Such an explanation need not evoke models of cell production and loss rate, or significant difference in cellcycle times, but only multiple tumour interaction, which has yet to be explained.

I would like to thank Professor P. J. Lindop for her help throughout this project, and William Hall for the diligent measurements of all these tumours. The work was carried out during the tenure of an M.R.C. grant and supported by grants from the British Empire Cancer Campaign for Research and the United States Public Health Service (Grant No. RH 00272).

### REFERENCES

BRUER, K.—(1965) Doctor of Medicine Thesis, University of Leiden.
COLLINS, V. P.—(1962) *Cancer, N.Y.*, **15**, 387.
COLLINS, V. P., LOEFFLER, R. K. AND TIVEY, H.—(1956) *Am. J. Roentg.*, **76**, 988.
DETHLEFSEN, L. A., PREWITT, J. M. S. AND MENDELSOHN, M. L.—(1968). *J. natn Cancer Inst.*, **40**, 389.
DU SAULT, L. A. AND KASENTER, A. G.—(1966) *Radiology*, **86**, 444.
HAWKES, M. J.—(1966) M.Phil. Thesis, University of London.
HAWKES, M. J., HILL, R. P., LINDOP, P. J., ELLIS, R. E. AND ROLBLAT, J.—(1968) *Br. J. Radiol.*, **41**, 134.
HILL, R. P.—(1967) Ph.D. Thesis, University of London.
LAIRD, A. K.—(1965) *Br. J. Cancer*, **19**, 278.
OWEN, L. N. AND STEEL, G. G.—(1969) *Br. J. Cancer*, **23**, 493.
PHILIPPE, E. AND LE GAL, Y.—(1968) *Cancer, N.Y.*, **21**, 461.
SCHWARTZ, M.—(1961) *Cancer, N.Y.*, **14**, 1272.
SPRATT, J. S. AND SPRATT, T. L.—(1964) *Ann. Surg.*, **159**, 161.
SPRATT, J. S., TER-POGASSIAN, M. AND LONG, T. L.—(1963) *Archs Surg., Chicago*, **86**, 283
STEEL, G. G., ADAMS, K. AND BARRETT, J. C.—(1966) *Br. J. Cancer*, **20**, 784.
STEEL, G. G. AND LAMERTON, L. F.—(1966) *Br. J. Cancer*, **20**, 74.
TANNOCK, I. F.—(1969) *Cancer Res.*, **29**, 1527.
WRIGHT, J. K. AND PETO, R. (1969) *Br. J. Cancer*, **23**, 547.